# Intraoperative Periprosthetic Fractures in Total Hip Arthroplasty: A 1.6-Million-Patient Analysis of Complications, Costs, and the Challenges in AI-Based Prediction

**DOI:** 10.3390/jcm13226862

**Published:** 2024-11-14

**Authors:** David Maman, Yaniv Steinfeld, Yaniv Yonai, Linor Fournier, Ofek Bar, Oleg Safir, Yaron Berkovich

**Affiliations:** 1 Department of Orthopedics, Carmel Medical Center, Haifa 3436212, Israel; yanivsteinfeld@gmail.com (Y.S.); yanivyonai@gmail.com (Y.Y.); linorfournier@gmail.com (L.F.); yaron.berkovich@gmail.com (Y.B.); 2 Technion Israel Institute of Technology, Haifa 2611001, Israel; 3 Faculty of Medicine, Lithuanian University of Health Sciences, 44307 Kaunas, Lithuania; ofekbar79@gmail.com; 4 Department of Orthopaedic Surgery, Mount Sinai Hospital, Toronto, ON M5G 1X5, Canada; oleg.safir@sinaihealth.ca; 5 Faculty of Medicine, University of Toronto, ON M5R 0A3, Canada

**Keywords:** total hip arthroplasty, big data, artificial intelligence, periprosthetic fracture

## Abstract

**Background:** Periprosthetic fractures following total hip arthroplasty are serious complications occurring in up to 2.4% of primary cases, contributing to significant morbidity, extended hospital stays, and elevated healthcare costs. Predicting these fractures remains a challenge despite advances in surgical techniques and prosthetic materials. **Methods:** This study analyzed 1,634,615 cases of primary THA from the NIS database (2016–2019) using propensity score matching to compare outcomes between patients with and without intraoperative periprosthetic fractures. Predictive models, including logistic regression, decision tree, and deep neural network, were evaluated for their ability to predict fracture risk. **Results:** Patients with periprosthetic fractures exhibited a 14-fold increase in pulmonary embolism risk, a 12-fold increase in infections, and a 5-fold increase in hip dislocations. Fractures extended hospital stays (3.8 vs. 2.5 days) and added approximately USD 32,000 in costs per patient. The predictive models yielded low accuracy (AUC max = 0.605), underscoring the complexity of predicting periprosthetic fractures. **Conclusions:** Intraoperative periprosthetic fractures in THA significantly elevate complication rates, costs, and length of stay. Despite extensive modeling efforts, accurate prediction remains difficult, highlighting the need to focus on preventive strategies, such as improved surgical techniques and real-time intraoperative monitoring.

## 1. Introduction

Intraoperative periprosthetic fractures during total hip arthroplasty (THA) are a significant and complex complication in orthopedic surgery, with reported incidence rates varying widely, reaching up to 2.4% in primary THA cases [1,2,3,4,5,6,7,8,9,10,11,12]. Additionally, various studies suggest that the actual number of cases may be higher, as many fractures go unreported [1,2,3,4,13,14,15]. These fractures present unique challenges, not only due to the mechanical disruption they cause, but also because of their association with a range of serious complications [16,17,18,19]. As the demand for THA continues to rise [10,20], driven by an aging population and the increasing prevalence of conditions such as osteoarthritis, understanding and addressing periprosthetic fractures has become a critical concern in the field of orthopedics.

The risk factors for periprosthetic fractures are multifactorial, with advanced age, osteoporosis, and previous fractures being among the most prominent [1,18]. The fractures in this study occurred intraoperatively, and their management requires complex and resource-intensive interventions, including revision surgery [21,22]. Beyond the immediate challenges of treatment, periprosthetic fractures are associated with prolonged recovery times, delayed rehabilitation, and increased dependence on healthcare resources [1,2,18,21].

The burden of periprosthetic fractures extends beyond clinical complications, significantly impacting both hospital resources and healthcare costs [1,2,18,20,21,23,24,25]. Patients who experience these fractures often face extended hospital stays, higher rates of readmission, and a greater need for postoperative care. These factors contribute to a substantial increase in healthcare expenditures, making periprosthetic fractures a costly complication for both patients and healthcare systems [1,7,8,9,18,23].

Despite advances in surgical techniques and prosthetic components, predicting and preventing periprosthetic fractures remains a significant challenge [26]. Current risk stratification models, which typically rely on patient demographics, comorbidities, and clinical history, have shown limited success in accurately identifying higher-risk individuals [26]. This underscores the need for more robust predictive tools and preventive strategies to mitigate the clinical and economic burden of periprosthetic fractures.

### Research Questions

This study explores both the clinical and economic impact of periprosthetic fractures during THA. By analyzing a large dataset, we seek to quantify the burden of these fractures in terms of hospital LOS, healthcare costs, and associated complications. Additionally, we assess the potential of artificial intelligence (AI) models to predict the occurrence of periprosthetic fractures, with the goal of improving risk stratification and guiding preventive measures. The findings of this research could provide valuable insights into the management of risk for periprosthetic fractures and contribute to the development of effective strategies to reduce their incidence and impact.

## 2. Methods

### 2.1. Dataset Acquisition and Inclusion Criteria

This study utilized a comprehensive dataset extracted from the Nationwide Inpatient Sample, the largest publicly available all-payer inpatient care database in the United States. The dataset encompassed 1,634,615 total cases of THA. The inclusion criteria focused on elective THA procedures identified through specific ICD-10 coding. The dataset spanned from 1 January 2016 to 31 December 2019, representing the most recent data available within the NIS system.

### 2.2. Patient Identification and Exclusions

Patients undergoing THA were identified using specific ICD-10 codes for total hip arthroplasty, ensuring that only elective procedures were included. Patients who underwent surgery before admission or had non-elective admissions were excluded to maintain the focus on elective cases. Additionally, cases reporting hospital costs of USD 0 were excluded from the analysis.

### 2.3. Statistical Analyses and Propensity Score Matching

Statistical analyses were conducted using SPSS 26 and MATLAB 2024, applying a significance level of *p* < 0.05. Comparative analyses between patients with and without intraoperative periprosthetic fractures were performed using crosstabs and independent-sample *t*-tests. To address potential selection bias and baseline differences between the groups, a propensity-score-matched analysis was employed. This approach created statistically comparable groups, ensuring that the comparison of outcomes, complications, and hospital characteristics was not confounded by baseline differences. The propensity-score-matched dataset included 12,905 patients with intraoperative periprosthetic fractures and an equal number of matched patients without fractures, accounting for factors such as age, gender, hospital location, and comorbidities.

### 2.4. Outcome Measures and Procedure Identification

Clinical outcomes, including in-hospital mortality, length of stay, complications, and overall hospitalization costs, were analyzed using established methodologies.

### 2.5. Intraoperative Periprosthetic Fracture Prediction Models

This study utilized a large dataset of more than 1.6 million patients who underwent THA to develop and evaluate models for predicting the likelihood of periprosthetic fractures. The dataset, which included 12,905 patients experiencing fractures, incorporated a wide range of clinical predictors. In total, 26 different predictors were used, covering demographics, comorbidities, and hospital characteristics. The dataset was randomly split into two subsets: 70% of the data (over 1.1 million patients) was used to train the models, while the remaining 30% (approximately 500,000 patients) was reserved for testing and evaluation. This approach ensured that the models were trained on a sufficiently large portion of the data while maintaining an independent test set for performance assessment. Three predictive models were developed and evaluated in this study: a logistic regression model, a decision tree model, and a deep neural network (DNN).

The logistic regression model was constructed using all 26 clinical predictors. This model aimed to predict the probability of periprosthetic fractures based on linear relationships between the predictors and the outcome. The decision tree model was designed to capture non-linear relationships between the predictors and the outcome. The decision tree was optimized for classification accuracy. Finally, a DNN was developed to capture complex patterns in the data. The DNN consisted of multiple layers and was trained on the 70% training set. To address the class imbalance, present in the dataset, class weighting was applied during training.

For each of the models, the key performance metrics, AUC, calibration intercept, and Brier Score were calculated to assess the model’s ability to discriminate between patients with and without periprosthetic fractures and the accuracy of the predicted probabilities. In addition to predicting periprosthetic fractures, we also applied the DNN model to predict the likelihood of blood transfusions as a reference. This additional analysis allowed us to compare the performance of the model in predicting another clinically relevant outcome, providing further context to the challenges faced in predicting periprosthetic fractures.

### 2.6. Ethical Aspects

The study was conducted under exempt status granted by the institutional review board, and the requirement for informed consent was waived due to the de-identified nature of the NIS dataset.

## 3. Results

A detailed comparative analysis of the dataset (1,634,615 patients, 2016 to 2019, obtained from the NIS) is presented in Table 1.

The average age at the time of surgery was 66.8 years for patients with intraoperative periprosthetic fractures, compared to 65.5 years for those without fractures. A significant difference was observed in the gender distribution, with a higher percentage of females in the intraoperative fracture group. Additionally, the percentage of patients treated in rural hospitals was higher in the intraoperative fracture group (10.7%) than in the no-fracture group (7.6%).

### 3.1. Analysis of Comorbidities in Patients with and Without Intraoperative Periprosthetic Fractures

As shown in Table 2, the intraoperative periprosthetic fracture group exhibited a higher prevalence of comorbidities compared to the non-fracture group. Specifically, as expected, the prevalence of osteoporosis was nearly twice as high in the fracture group (8.8%) compared to the non-fracture group (4.6%), underscoring the known relationship between osteoporosis and fracture risk during surgery. Additionally, other comorbidities such as chronic lung disease, hypertension, and type 2 diabetes mellitus were also significantly more common in patients who sustained intraoperative fractures, suggesting that these conditions may contribute to an increased risk of this serious complication.

### 3.2. Propensity-Score-Matched Analysis

In order to overcome potential selection bias and baseline differences in terms of existing comorbidities, a propensity-score-matched analysis was performed, ensuring that the two groups compared were statistically equivalent, thus minimizing selection bias. Propensity-score-matched analysis is a statistical method that helps compare two groups in observational studies. It balances participant characteristics by pairing individuals with similar likelihoods of being in either group, making the comparison more reliable and reducing the impact of confounding variables. This approach aims to emulate the random assignment seen in experiments, improving the accuracy of conclusions drawn from non-randomized studies. It offers insights into demographics, payer details, and the prevalence of various medical conditions, demonstrating the average age, gender distribution, and type of payer, alongside an array of diagnoses, in both the intraoperative periprosthetic fracture and non-fracture groups. The propensity-score-matched analysis data are presented in Table 3. No significant disparities were discerned between the groups in terms of the examined parameters, highlighting the homogeneous nature of the two patient cohorts and underlining the effectiveness and reliability of the applied propensity-score-matched analysis.

### 3.3. Comparison of Length of Stay and Total Charges Between Patients with and Without Intraoperative Periprosthetic Fracture in Propensity-Score-Matched Cohorts

A detailed comparison of the length of stay and total charges between patients who experienced intraoperative periprosthetic fractures and those who did not is presented in Table 4. Patients with intraoperative periprosthetic fractures had a significantly longer hospital stay, averaging 3.8 days compared to 2.5 days for those without fractures. Additionally, the total charges were considerably higher for patients with fractures, with an average of USD 93,184 compared to USD 60,413 for patients without fractures.

### 3.4. Odds Ratios of Complications in Patients with Intraoperative Periprosthetic Fractures Compared to Those Without Fractures

**Figure 1:**
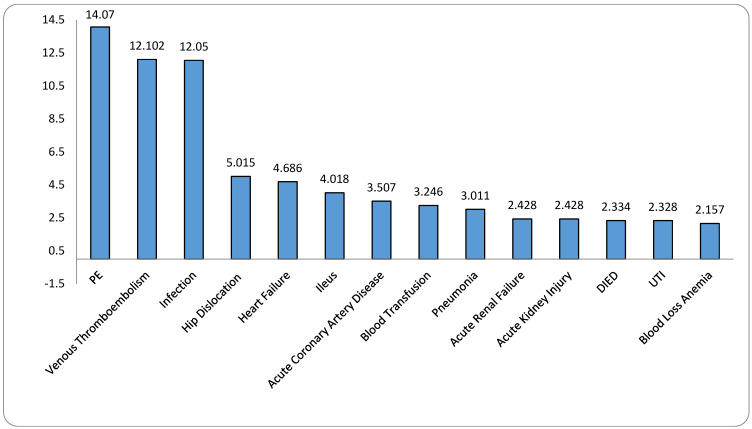
Odds ratios of complications in patients with intraoperative periprosthetic fractures compared to those without fractures.

Figure 1 presents the odds ratio (OR) with 95% confidence interval (CI) for various postoperative complications in patients who experienced intraoperative periprosthetic fractures compared to those without fractures. The data highlight significantly higher risks associated with these fractures, with all *p*-values being less than 0.0001.

PE stands out with the highest risk, showing an OR of 14.07 (95% CI: 5.677–34.871), indicating a markedly increased likelihood of occurrence in patients with fractures. Venous thromboembolism also demonstrates a substantial elevation in risk, with an OR of 12.102 (95% CI: 6.346–23.08). Infection exhibits an OR of 12.05 (95% CI: 4.838–30.016).

The risk of hip dislocation is significantly higher in this group, with an OR of 5.015 (95% CI: 2.542–9.893). Heart failure shows an elevated risk as well, with an OR of 4.686 (95% CI: 2.682–8.189). Ileus has an OR of 4.018 (95% CI: 2.46–6.563). Acute coronary artery disease presents with an OR of 3.507 (95% CI: 1.736–7.084), and the need for blood transfusion with an OR of 3.246 (95% CI: 2.962–3.558). Pneumonia shows an OR of 3.011 (95% CI: 1.914–4.738). Acute renal failure has an OR of 2.428 (95% CI: 2.116–2.785). The odds of mortality are also elevated, with an OR of 2.334 (95% CI: 1.274–4.276). Urinary tract infection (UTI) has an OR of 2.328 (95% CI: 1.899–2.854), and blood loss anemia is more prevalent in this group, with an OR of 2.157 (95% CI: 2.042–2.279).

### 3.5. Intraoperative Periprosthetic Fracture Prediction Models

The performance of three predictive models—logistic regression, decision tree, and deep neural network (DNN)—was evaluated using AUC, calibration intercept, and Brier Score. As shown in Table 5 the results from these metrics underscore the significant challenges in accurately predicting intraoperative periprosthetic fractures. The logistic regression model achieved an AUC of 0.605, indicating a modest ability to discriminate between patients with and without fractures. However, the model’s calibration intercept of −5.4587 suggests that it generally underestimated the probability of fractures. The Brier Score of 0.00773 indicates reasonable accuracy in probability predictions, but this must be interpreted with caution given the model’s calibration issues.

The decision tree model performed poorly, with an AUC of 0.500, suggesting no better predictive power than random guessing. The calibration intercept for this model was −4.844, further indicating that the model tended to underestimate fracture risk. The Brier Score of 0.009 reflects lower accuracy in probability predictions compared to the logistic regression model. The DNN model produced an AUC of 0.601, similar to that of the logistic regression model. However, like the other models, it faced calibration challenges, with a calibration intercept of −4.845. The Brier Score of 0.008 indicates a comparable level of accuracy in probability predictions to the logistic regression model.

For reference, we also applied the DNN model to predict blood transfusions, which yielded a significantly higher AUC of 0.820, indicating better predictive performance in this context. The calibration intercept for the blood transfusion model was −0.50, and the Brier Score was 0.120, reflecting improved accuracy and calibration compared to the fracture models. Overall, these results demonstrate that all three models, despite varying levels of complexity, struggled to accurately predict intraoperative periprosthetic fractures. The modest AUC values and negative calibration interceptions across all models suggest that, even with comprehensive data and advanced algorithms, predicting fractures remains a significant challenge in clinical practice.

## 4. Discussion

Main Findings

This study highlights the significant clinical and economic impact of intraoperative periprosthetic fractures following THA. Patients with periprosthetic fractures demonstrated substantially higher rates of postoperative complications, including a 14-fold increase in PE, a 12-fold increase in infections, and a 5-fold increase in hip dislocations compared to those without fractures. These fractures were also associated with prolonged hospital stays and significantly higher healthcare costs, averaging an additional USD 32,000 per patient. Despite the use of advanced AI models applied to a dataset of over 1.6 million patients, predicting which individuals are at risk for these fractures remains a major challenge, reflecting the complexity of the issue.

Clinical Implications of Periprosthetic Fractures and Dislocations

Periprosthetic fractures have far-reaching clinical implications, extending beyond immediate implant failure. The association between these fractures and complications such as DVT, PE, and infections is well documented [1,27]. Our study further confirms that hip dislocations are significantly more common in patients with periprosthetic fractures, raising concerns that some fractures may go undetected during surgery, compromising implant stability [2]. The potential for detected and undetected fractures to lead to misalignment and subsequent dislocation has been suggested in prior research, where subtle fractures were found to destabilize implants [12,27,28,29,30,31,32,33,34,35,36].

Given these risks, improving the intraoperative detection of fractures is crucial. Techniques such as intraoperative fluoroscopy and advanced monitoring systems could play a critical role in identifying fractures that might otherwise go unnoticed. Additionally, developing surgical techniques and implants tailored to address the challenges posed by periprosthetic fractures may reduce the risk of postoperative complications.

Economic Burden and Healthcare Resource Utilization

The economic impact of periprosthetic fractures is substantial. Previous studies have shown that managing these fractures incurs significant costs, largely due to revision surgeries, extended hospital stays, and intensive postoperative care [11]. Our findings support this, showing that patients with periprosthetic fractures incur healthcare costs that are approximately USD 32,000 higher per patient compared to those without fractures. This financial burden is further amplified by the increased likelihood of complications, which drive up both costs and resource utilization [19,27,31,32,33].

The prolonged hospital stays observed in this study align with earlier research, which has shown that periprosthetic fractures often require more complex and resource-intensive care. Beyond direct hospital costs, patients with these fractures are also more likely to need long-term rehabilitation and outpatient care, further exacerbating the financial impact [1,4,19]. These findings underscore the necessity of developing cost-effective preventive strategies and optimizing care pathways to reduce the economic burden associated with periprosthetic fractures.

### 4.1. Challenges in Predicting Periprosthetic Fractures

Accurately predicting which patients are at risk for periprosthetic fractures remains a significant challenge despite advances in AI and machine learning. In this study, predictive models, including logistic regression, decision trees, and deep neural networks, showed only modest performance, with the highest AUC reaching 0.605. This is consistent with other studies that have struggled to achieve high predictive accuracy for periprosthetic fractures [18,19].

The challenge of prediction stems from the multifactorial nature of periprosthetic fractures. Factors such as patient demographics, comorbidities, bone quality, surgical technique, and implant characteristics all contribute to fracture risk. However, many of these factors are not fully captured in large administrative datasets like the NIS, limiting the ability of predictive models to consider all relevant variables [17,27,31]. For instance, while bone mineral density and cortical defects are known predictors of periprosthetic fractures, such data are often missing from large-scale datasets. Our focus on preoperative data, aiming to identify at-risk patients before surgery, adds another layer of complexity since detailed information about bone quality and cortical defects is typically unavailable prior to surgery. This lack of critical preoperative data hampers the accuracy of risk stratification and highlights the need for improved preoperative assessments or new methods to obtain relevant information before surgery.

The better performance of our models in predicting blood transfusion underscores the challenges in predicting periprosthetic fractures. Blood transfusion prediction relies on clear preoperative factors such as chronic anemia, which contrasts with the complex and multifactorial nature of periprosthetic fractures. This discrepancy highlights the need for enhanced preoperative assessments and better surgical techniques, or the development of new methods to capture relevant information before surgery to improve fracture risk prediction.

### 4.2. Limitations

Several limitations of this study must be acknowledged. The use of the NIS database, while providing a large and diverse dataset, introduces potential challenges related to coding accuracy and data completeness. Administrative databases like the NIS often lack detailed clinical information, such as bone quality, surgical technique, and intraoperative events, which are critical for understanding the full scope of periprosthetic fracture risk [37,38,39,40]. This limitation may have affected the ability of the AI models to accurately predict fracture risk. Although propensity score matching was employed to mitigate selection bias, unmeasured confounders may still be present. For example, surgeon experience and hospital volume, which have been shown to influence outcomes in orthopedic surgery, were not accounted for in the analysis. Future studies should consider incorporating these factors to provide a more comprehensive assessment of fracture risk [37,38,39,40]. Additionally, the incidence rate for periprosthetic fractures, based on ICD-10 coding in the NIS database, may indeed be lower than the true incidence due to factors such as underreporting, coding errors, or fractures that were only discovered after discharge [1,2,18,19].

## 5. Conclusions

This study underscores the significant clinical and economic burden of periprosthetic fractures in patients undergoing THA. Despite the use of a large dataset and advanced AI models, predicting these fractures remains a formidable challenge. The increased risk of complications, particularly hip dislocations, may suggest that some fractures are undetected during surgery, leading to instability, stem subsidence, and subsequent dislocation. These findings highlight the need for continued innovation in surgical practices to reduce the incidence and impact of periprosthetic fractures.

Future research should focus not only on refining predictive models, but also on acknowledging the current limitations in accurately predicting periprosthetic fractures. Given the challenges in prediction, the emphasis should shift towards preventing these fractures through the development and implementation of new and improved technologies that can quantifiably monitor bone changes in real time during the implant insertion phases of THA. The insights gained from this study emphasize the need for innovation in surgical strategies as the primary means of addressing this complex and costly complication in orthopedic surgery.

## Figures and Tables

**Table 1:** Comparative analysis of intraoperative and no intraoperative periprosthetic fractures by age, payer distribution, gender, and hospital type in THA.

Parameter	No Intraoperative Periprosthetic Fracture	Intraoperative Periprosthetic Fracture	Significance
Average age (y)	65.5	66.8	*p* < 0.0001
Female (%)	55.3%	65.3%	*p* < 0.0001
Primary expected payer—Medicare (%)	54.6%	60.9%	*p* < 0.001
Primary expected payer—Medicaid (%)	5.0%	7.7%	*p* < 0.001 *p* < 0.001
Primary expected payer—private, including HMO (%)	37.2%	28.2%	
Primary expected payer—self-pay (%)	0.7%	1.0%	
Primary expected payer—no charge (%)	0.1%	0.1%	
Primary expected payer—other (%)	2.4%	2.1%	
Location/teaching status of hospital (STRATA)—Rural (%)	7.6%	10.7%	
Location/teaching status of hospital (STRATA)—Urban nonteaching (%)	24.8%	22.7%	*p* < 0.001
Location/teaching status of hospital (STRATA)—Urban teaching (%)	67.6%	66.6%	

**Table 2:** Comparative analysis of comorbidities between intraoperative and no intraoperative periprosthetic fracture groups in THA.

Parameter	No Intraoperative Periprosthetic Fracture	Intraoperative Periprosthetic Fracture	Significance
Hypertension	52.1%	54.1%	*p* < 0.001
Dyslipidemia	42.3%	42.7%	*p* = 0.401
Obstructive Sleep Apnea	10.1%	9.3%	*p* = 0.005
Chronic Anemia	5.7%	7.9%	*p* < 0.001
Alcohol Abuse	1.5%	2.5%	*p* < 0.001
Osteoporosis	4.6%	8.8%	*p* < 0.001
Type 2 Diabetes Mellitus	14.9%	17.0%	*p* < 0.001
Chronic Kidney Disease	6.4%	8.9%	*p* < 0.001
Congestive Heart Failure	1.2%	2.1%	*p* < 0.001
Chronic Lung Disease	6.6%	10.2%	*p* < 0.001
History of Myocardial Infarction	3.4%	4.4%	*p* < 0.001
History of Cerebrovascular Accident	3.8%	5.5%	*p* < 0.001
Dementia	0.5%	1.5%	*p* < 0.001
Neoplasms	1.4%	1.5%	*p* = 0.123
Neoplasms of Lymphoid and Hematopoietic Tissue	0.6%	0.5%	*p* = 0.152
Peripheral Vascular Disease	1.7%	2.0%	*p* = 0.010
BMI < 30	76.5%	75.9%	*p* < 0.001
Obesity	11.2%	10.3%	*p* < 0.001
Morbid Obesity	12.2%	13.8%	*p* < 0.001

**Table 3:** Comparison of demographic and clinical data in propensity-score-matched cohorts of patients with and without intraoperative periprosthetic fractures.

Parameter	No Intraoperative Periprosthetic Fracture	Intraoperative Periprosthetic Fracture	Significance
Total Surgeries (Number)	12,905	12,905	
Average Age (Years)	66.8	66.8	*p* = 0.90
Female (%)	65.4	65.3	*p* = 0.89
Payer—Medicare (%)	61.3	60.9	*p* = 0.06
Payer—Medicaid (%)	7.5	7.7	
Payer—Private (%)	28.4	28.2	
Payer—Self-pay (%)	0.7	1.0	
Payer—No charge(%)	0.0	0.1	
Payer—Other (including Self-pay) (%)	2.1	2.1	
Hypertension Diagnosis (%)	54.2	54.1	*p* = 0.80
Dyslipidemia Diagnosis (%)	43.1	42.7	*p* = 0.53
Sleep Apnea Diagnosis (%)	9.5	9.3	*p* = 0.59
Chronic Anemia (%)	7.3	7.9	*p* = 0.08
Alcohol Abuse (%)	2.4	2.5	*p* = 0.55
Osteoporosis (%)	8.9	8.8	*p* = 0.83
Type 2 Diabetes (%)	17.2	17.0	*p* = 0.68
Renal Disease (%)	8.8	8.9	*p* = 0.66
Chronic Heart Failure (%)	2.1	2.1	*p* = 0.65
Chronic Lung Disease (%)	10.3	10.2	*p* = 0.68
History of MI (%)	4.2	4.4	*p* = 0.07
Peripheral Vascular Disease (%)	2.1	2.0	*p* = 0.51
History of CVA (%)	5.3	5.5	*p* = 0.07
Neoplasms (%)	1.3	1.5	*p* = 0.07
Neoplasms (Lymphoid/Hematopoietic) (%)	0.6	0.5	*p* = 0.09
BMI < 30	75.8	75.9	*p* = 0.09
Obesity	10.5	10.3	
Morbid Obesity	13.7	13.8	

**Table 4:** Comparison of hospitalization outcomes in propensity-score-matched cohorts between intraoperative and no intraoperative periprosthetic fracture groups in THA.

Parameter	No Intraoperative Periprosthetic Fracture	Intraoperative Periprosthetic Fracture	Significance
Length of stay mean in days	2.5 (Std. deviation 1.7)	3.8 (Std. deviation 6.0)	*p* < 0.0001
Total charges mean in USD	60,413 (Std. deviation 32,800)	93,184 (Std. deviation 78,611)	*p* < 0.0001

**Table 5:** Performance of intraoperative periprosthetic fracture prediction models and blood transfusion for reference.

Metric	Logistic Regression (Periprosthetic Fracture)	Decision Tree (Periprosthetic Fracture)	Deep Neural Network (Periprosthetic Fracture)	Deep Neural Network (Blood Transfusions)
AUC (Test Set)	0.60	0.50	0.60	0.820
Calibration Intercept (Test Set)	−5.46	−4.84	−4.86	−0.50
Brier Score (Test Set)	0.008	0.009	0.008	0.120

## Data Availability

The original contributions presented in the study are included in the article/Supplementary Materials; further inquiries can be directed to the corresponding author.

## Supplementary Materials

The following supporting information can be downloaded at https://www.mdpi.com/article/10.3390/jcm13226862/s1: Table S1: Icd10 codes.

## Abbreviations

AUC	Area under the curve
HCUP	Healthcare Cost and Utilization Project
IBD	Inflammatory bowel disease
DNN	Deep neural network
ICD-10	International Classification of Diseases, 10th Revision
LOS	Length of stay
NIS	Nationwide Inpatient Sample
SPSS	Statistical Package for the Social Sciences
THA	Total hip arthroplasty
