# Supplementary material for: Intraoperative Periprosthetic Fractures in Total Hip Arthroplasty: A 1.6-Million-Patient Analysis of Complications, Costs, and the Challenges in AI-Based Prediction
Source: J Clin Med. 2024 Nov 14;13(22):6862. doi: 10.3390/jcm13226862 (PMC11594302; doi:10.3390/jcm13226862)
Supplement: Supplementary file 1 [file jcm-13-06862-s001.zip › jcm-3306795-supplementary.pdf]

ICD 10 CODES / PROCEDURE CODE	
0QS706Z,0QS606Z,0QS704Z,0QS604Z,0QS736Z,0QS636Z,0SRS0JZ,0SRR0JZ, 0SRS0J9,0SRS0JA,0QS734Z,0SRR0J9,0QS634Z,0SRR0JA,0QH734Z,0SRS01A, 0QH634Z,0SRR01A,0SRS019,0SRR019,0QH706Z,0QH606Z, 0QH736Z,0QH636Z,0SRR01Z,0SRS01Z,0QH704Z,0QH604Z,0SRB0JZ,0SR90JZ,0SR904A, 0SRB04A,0SRB0JA,0SR90JA,0SR902A,0SRB02A,0SRS039,0SR904Z,0SRB04Z,0SRR039,0SRS03A, 0SR90J9,0SRR03A,0SRB0J9,0SR902Z,0SRB02Z,0SRS03Z	Total hip arthroplasty procedure
I5021, I5031, I5033, I5041, I5043	Heart Failure
N170, N171, N172, N178, N179	Acute Kidney Injury
I2101, I2102, I2109, I211, I2119, I2111, I212, I2129, I213, I214, I219	Acute Coronary Artery Disease
I60, I61, I62, I63, I650, I688, O873, O2250, O2251, O2252	Stroke
J810, J811, I501	Pulmonary Edema
I10(start with)	Hypertension
D62 (start with)	Blood Loss Anemia
J189, J159, J22	Pneumonia
I2602, I2609, I2692, I2699	Pulmonary Embolism
I82401, I82402, I82403, I82409, I82411, I82412, I82413, I82419, I82421, I82422, I82423, I82429	DVT
E78(start with)	Dyslipidemia
G473	Obstructive Sleep Apnea
D64(start with)	Chronic Anemia
F10	Alcohol Abuse History
M81, M82	Osteoporosis
F (start with)	Mental Disorders
G20 (start with)	Parkinson Disease
E11 (start with)	Type 2 Diabetes Mellitus
N18 (start with)	Chronic Kidney Disease
I500, I501, I509	Congestive Heart Failure
J44 (start with)	Chronic Lung Disease
